# Case Report: Systemic treatment for breast and vulvar metastases from resected rectal signet ring cell carcinoma

**DOI:** 10.3389/fonc.2023.1213888

**Published:** 2023-07-06

**Authors:** Yihui Han, Wenming Yang, Qin Ma, Zhaolun Cai, Yun Yang, Junhe Gou, Tao Yuan, Mingming Zhang, Bo Zhang

**Affiliations:** ^1^ Department of General Surgery, West China Hospital, Sichuan University, Chengdu, China; ^2^ Gastric Cancer Center, West China Hospital, Sichuan University, Chengdu, China; ^3^ Division of Gastrointestinal Surgery, Department of General Surgery, West China Hospital, Sichuan University, Chengdu, China; ^4^ Department of General Surgery, West China Shangjin Hospital, Sichuan University, Chengdu, China; ^5^ Colorectal Cancer Center, West China Hospital, Sichuan University, Chengdu, China; ^6^ Department of Pathology, West China Shangjin Hospital, Sichuan University, Chengdu, China; ^7^ Department of Anesthesiology, West China Shangjin Hospital, Sichuan University, Chengdu, China

**Keywords:** rectal signet ring cell carcinoma, breast metastasis, vulvar metastasis, systemic treatment, immunohistochemical staining, mismatch repair, microsatellite stability

## Abstract

**Background:**

Breast and vulvar metastases from rectal signet ring cell carcinoma (SRCC) represent a rare and obscure clinical entity associated with poor survival. Managing patients with metastatic rectal SRCC is extremely challenging due to the absence of high-quality evidence.

**Case presentation:**

A 26-year-old woman presented with progressively worsening anal pain, constipation, and hematochezia for approximately two years. Following the diagnosis of locally advanced rectal cancer (_c_T_3_N_0-1_M_0_), she received neoadjuvant chemotherapy with modified FOLFOX6 regimen and underwent laparoscopic abdominoperineal resection. Metastases to the breast and vulva developed during postoperative chemotherapy. Genetic testing revealed RAS/BRAF wild-type and microsatellite instability (MSI)-low status. Though sequential administration of irinotecan plus tegafur and tegafur plus raltitrexed-based chemotherapy in combination with bevacizumab, the disease progressed rapidly. Sadly, the patient passed away 15 months after initial diagnosis due to rapidly progressive disease.

**Conclusion:**

Rectal SRCC is associated with younger on-set, aggressive behaviors, and worse survival outcomes. Due to poor cohesiveness, SRCC tends to develop metastases. A patient’s medical history and immunohistochemical staining (such as CK20, CK7, and CDX-2) can aid in identifying the tumor origin of breast and vulvar metastases. Mutations and signaling pathways predominant in the tumorigenesis of SRCC remains unveiled. There is poor effect of conventional chemotherapies, targeted and immunotherapies for colorectal adenocarcinoma on SRCC, so novel therapies are needed to treat this patient population.

## Introduction

Colorectal cancer (CRC) is the third most commonly diagnosed malignancy and the second leading cause of cancer-related deaths worldwide (1). Signet ring cell carcinoma (SRCC), characterized by the presence of over 50% signet ring cells, represents one of the rarest histopathologic subtypes of CRC (1-2.4%) and is associated with poorer survival outcomes (2, 3). Signet ring cells are featured by an eccentrically displaced nucleus due to excess intracellular mucin, imparting a signet ring appearance under an optical microscope (4). Although the median age at CRC diagnosis is 63-69 years (5), colorectal SRCC is associated with a younger on-set (6). Due to poor cohesiveness, SRCC tends to develop metastases (7). Distant metastases from colorectal SRCC are most frequently found in the liver, followed by the distant lymph node, bone, lung, and brain (8).

Most breast metastases originate from the contralateral breast cancer (9). Breast metastases from extramammary malignancies are exactly uncommon, with incidence rates ranging between 0.2% and 2.7% in previous studies (10, 11). Except CRC, hematological malignancies as well as several primary solid tumors (including melanoma, rhabdomyosarcoma, and lung cancer) usually disseminate to the breast (12). Similarly, accounting for only 4% of gynecological malignancies, primary vulvar cancer is uncommon and mainly affects postmenopausal women (13). Vulvar metastasis is even rarer, representing only 5-8% of all vulvar cancers (14, 15).

In accordance with principles of the CAse REport (CARE) guidelines (16), we present a case report of a young female patient with breast and vulvar metastases from resected rectal SRCC. This case report aims to enhance the clinicians’ recognition and awareness of the invasiveness and occult metastasis of colorectal SRCC.

## Case presentation

**Figure 1:**
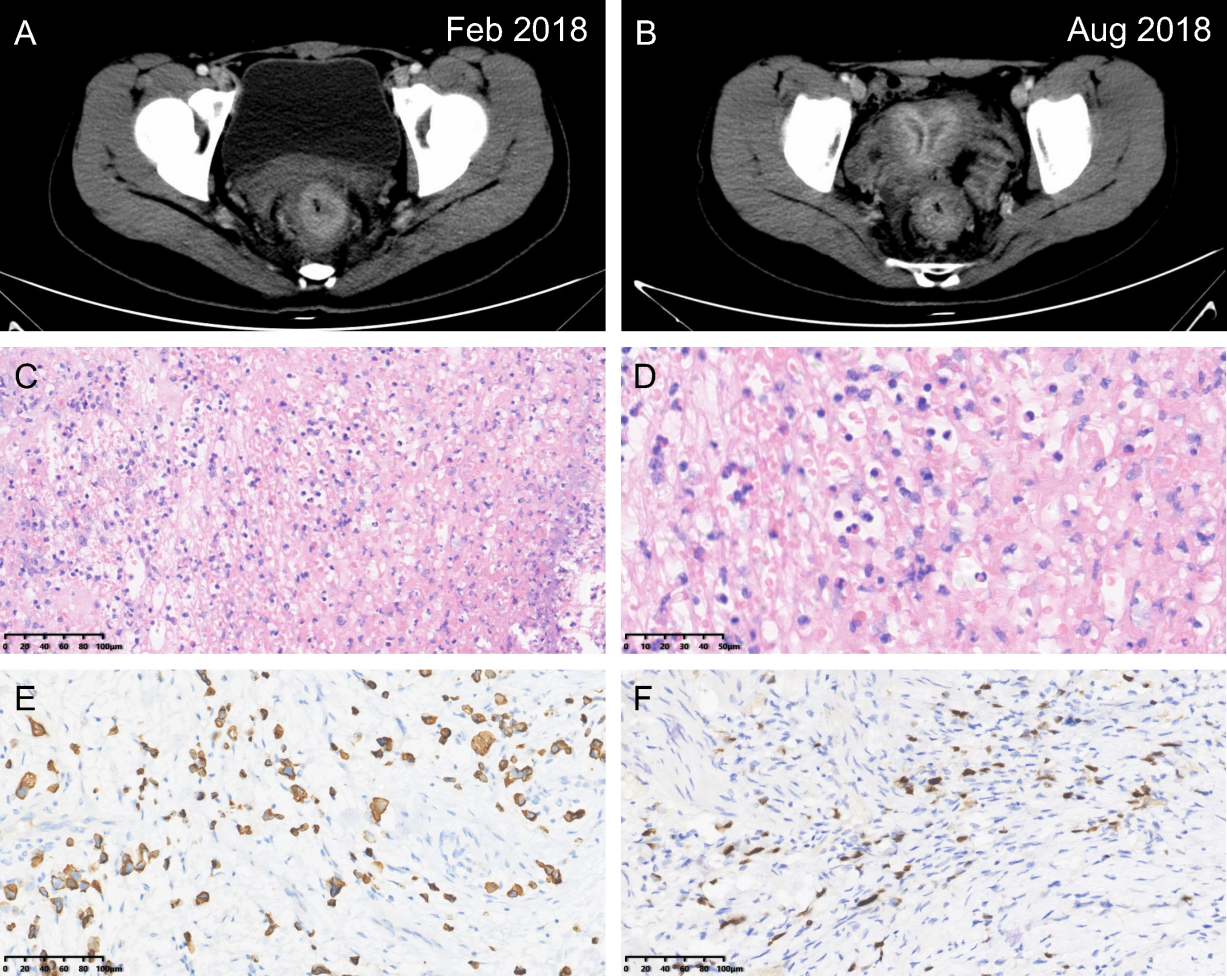
Rectal signet ring cell carcinoma. Computed tomography images at the initial diagnosis **(A)** and after the implementation of neoadjuvant therapy **(B)**. Hematoxylin & eosin staining imparting a signet ring appearance (**C**, magnification power: 20×; **D**, magnification power: 40×). Immunohistochemical staining revealing **(E)** CK20 (+), and **(F)** CDX-2 (+) (magnification power: 20×).

In February 2018, a 26-year-old female patient presented to our hospital with progressively worsening anal pain, constipation, and hematochezia for approximately two years. She had given birth right two and a half years previously. She had no prior medical history and family history of gastrointestinal malignancies. On admission, her vital signs were stable. Digital rectal examination revealed an ulcerated 5-cm indurated lesion located at the nine o’clock position and 2 cm from the anal verge, with bleeding upon palpation. The blood tests showed elevated carcinoembryonic antigen (CEA) (10.51 ng/ml; reference range, < 3.4 ng/ml) and carbohydrate antigen 19-9 (CA19-9) (40.49 U/ml; reference range, < 22 U/ml). The diagnosis of poorly differentiated adenocarcinoma was established by flexible sigmoidoscopy with biopsies and histological examination. Computed tomography (CT) of the chest and abdomen and magnetic resonance imaging (MRI) of the pelvic confirmed the tumor stage of cT_3_N_1_M_0_ (**Figure 1A**). Radiologic examinations did not detect metastasis to the liver, bone, lung, or breast.

Following consultancy with the multidisciplinary team, the patient underwent prophylactic transverse colostomy to avoid upcoming obstruction and then received 6 cycles of modified FOLFOX-6 neoadjuvant chemotherapy. The scheduled long-course radiotherapy (50Gy/25f) was ceased after the first time due to severe anal incontinence and myelosuppression. Laparoscopic abdominoperineal resection (R0) with a permanent colostomy was performed for her 6 weeks after the termination of neoadjuvant therapy (**Figure 1B**). Guided by the fast-track surgery pathway, the patient’s recovery was uneventful, with discharge on postoperative day 5. The final diagnosis of rectal SRCC (ypT_3_N_1b_M_0_, Tumor Regression Grade 2) was determined via postoperative pathologic findings (**Figures 1C–F**). Two out of eighteen mesorectal lymph nodes were identified with tumor involvement. Meanwhile, the proficient mismatch repair (MMR) was detected. The postoperative chemotherapy was consistent with the neoadjuvant regimen and initiated 4 weeks after surgery.

**Figure 2:**
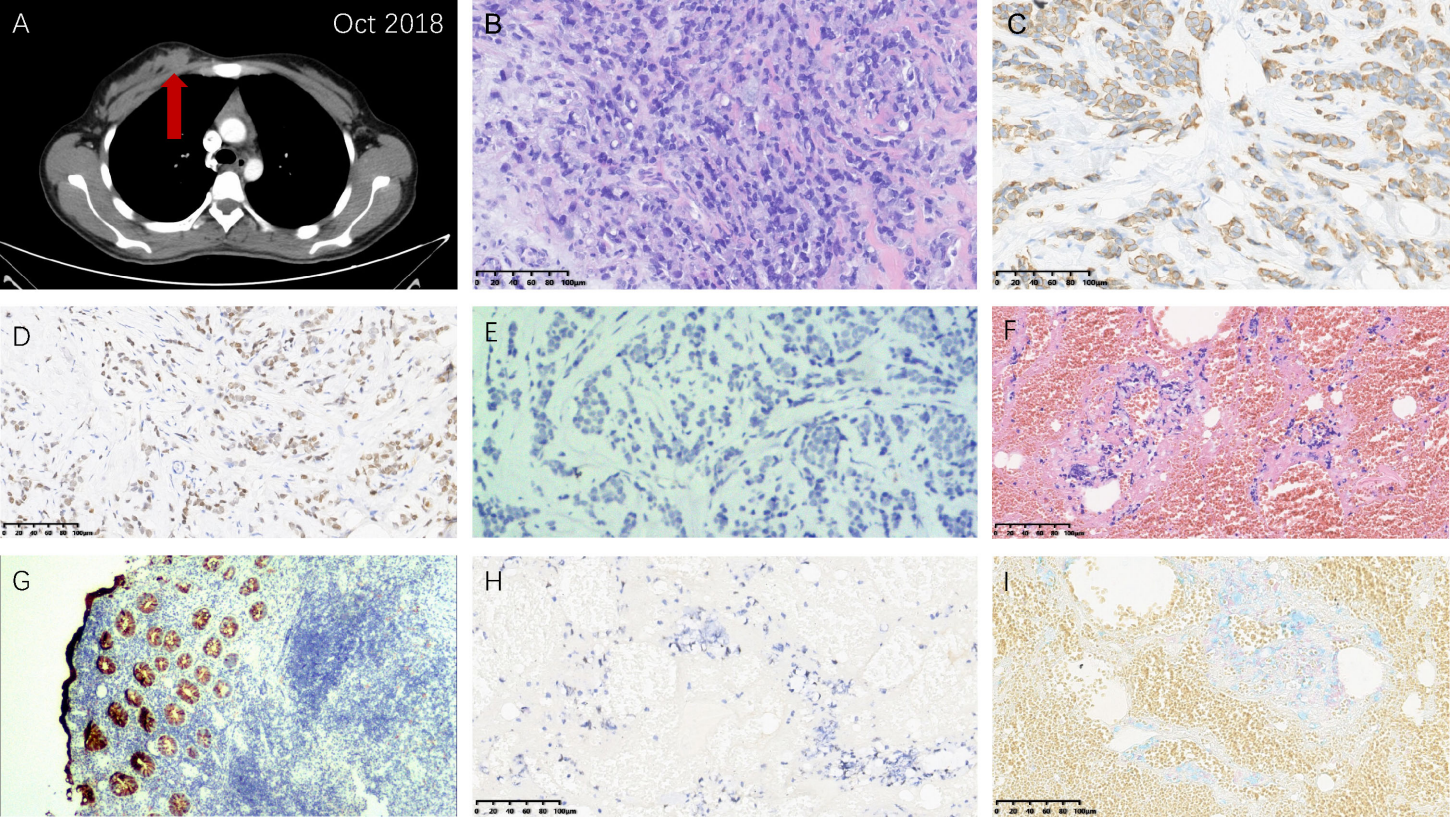
Right breast and vulvar metastases from rectal signet ring cell carcinoma. **(A)** Computed tomography image showing the right breast metastasis. Hematoxylin & eosin staining demonstrating morphological characteristics of **(B)** the right breast and **(F)** vulvar metastases similar with the primary rectal carcinoma (magnification power: 20×). Immunohistochemical staining: **(C)** CK20 (+) (magnification power: 20×), **(D)** CDX-2 (+) (magnification power: 20×), and **(E)** GATA3 (-) (magnification power: 100×) of the right breast metastasis tissue; **(G)** CK20 (+) (magnification power: 40×), **(H)** CDX-2 (+) (magnification power: 20×), and **(I)** Alcian Blue (+) (magnification power: 20×) of the vulvar metastasis tissue.

After the second cycle of adjuvant modified FOLFOX-6, a painless, firm mass was palpable in the right breast and the area of vulva, respectively. The follow-up CT examination found the right breast mass (**Figure 2A**). Ultrasound-guided core needle biopsies were performed for her. Hematoxylin & eosin and immunohistochemical (IHC) staining indicated the metastases to the breast (positive: CK20, CDX-2, E-cadherin; negative: CK7, PR, ER, Her-2, GATA3, GCDFP15) (**Figures 2B–E**) and vulva (positive: CDX-2, PCK, CEA, Alcian Blue) (**Figures 2F–I**) from rectal SRCC. In addition, genetic testing demonstrated RAS/BRAF wild-type and microsatellite instability-low (MSI-L).

**Figure 3:**
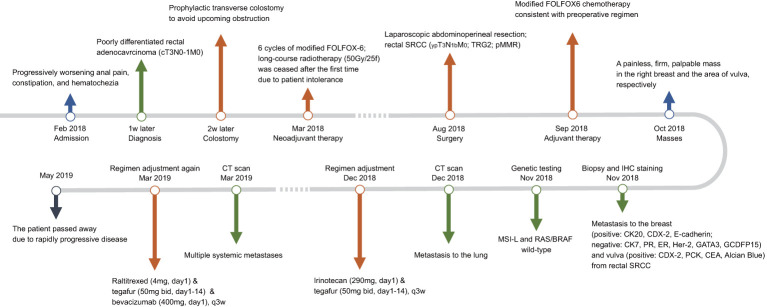
Timeline with clinical data from the episode of care. SRCC, signet ring cell carcinoma; TRG2, tumor regression grade 2; pMMR, proficient mismatch repair; IHC, immunohistochemical; MSI-L, microsatellite instability-low; CT, computed tomography.

In December 2018, metastases to bilateral lung have been developed and the evaluation of efficacy was identified as progressive disease. Then the chemotherapy regimen was changed to irinotecan (290mg, day1, q3w) plus tegafur (50mg bid, day1-14, q3w). However, multiple metastases throughout the body (including the left breast) were found 3 months later. Tegafur (50mg, bid, day1-14, q3w) plus raltitrexed (4mg, day1, q3w)-based chemotherapy in combination with bevacizumab (400mg, day1, q3w) as the third-line treatment did not provide favorable efficacy. Unfortunately, the patient passed away 15 months after initial diagnosis due to rapidly progressive disease. The results of serum tumor markers (CEA and CA19-9) are listed in **Table 1**. Though limited sensitivity, the reduction of tumor markers at the early phase represented favorable response to neoadjuvant therapy, while the elevation of tumor markers at the late phase indicated rapidly progressive disease. The timeline with clinical data from the episode of care is shown in **Figure 3**.

**Table 1:** Serum tumor markers (CEA and CA19-9) of the patient during the episode of care.

Time	CEA (ng/ml) (Ref. < 3.4)	CA19-9 (U/ml) (Ref. < 22)
Feb 2018	10.51	40.49
Mar 2018	20.97	74.90
Apr 2018	10.89	77.64
Jun 2018	3.04	53.66
Jul 2018	2.55	63.21
Aug 2018	2.03	77.25
Sep 2018	1.55	46.88
Nov 2018	1.13	40.54
Dec 2018	2.12	47.17
Jan 2019	3.65	64.59
Feb 2019	15.15	198.9
Mar 2019	46.15	312.3

CEA, carcinoembryonic antigen; CA19-9, carbohydrate antigen 19-9.

## Discussion

Compared to the liver or lung metastases, rectal cancer breast metastasis (RCBM), let alone vulvar metastasis, is rather uncommon in existing literature (17–19). Except 2 male patients, RCBM mainly involved the female in previous reports (20, 21). The rareness of this entity might be ascribed to its hormone status and the relatively poor blood supply of a large amount of fibrous tissue, especially in Eastern female (22, 23). Besides hematogenous spread, lymphatic dissemination of cancer cells to thoracic duct might play a critical role in RCBM (24). RCBM presents at a younger age of 47.7 years (17) than primary rectal cancer (63 years) (5). The mean interval between the initial diagnosis of rectal cancer and the occurrence of breast metastasis was reported to be 28.4 months (range, 5 months to 18 years) (17). Williams et al. (25) suggested that breast metastasis usually served as a part of systemic metastases and was associated with poor expected survival, with a median survival of 10 months post diagnosis. Therefore, a thorough examination to evaluate metastases to other sites is required.

The most essential evidence to help establish the diagnosis of RCBM are the past medical history and morphological assessment of hematoxylin and eosin-stained sections in comparison to the primary tumor (10). Furthermore, IHC using specific primary antibodies remains the golden standard for the diagnosis of RCBM. The combination of cytokeratin 20 (CK20) and cytokeratin 7 (CK7) is of great benefit to the categorization of carcinomas and discrimination of primary adenocarcinomas from metastatic ones (26, 27). Meanwhile, caudal type homeobox 2 (CDX-2) is only expressed by intestine cells in adults and can be also employed to determine the colorectal adenocarcinoma (28). Conversely, GATA binding protein 3 (GATA3) is highly expressed in well-differentiated primary breast cancer (29). When conducting IHC assay, panels of antibodies, rather than any individuals, should be relied on (10).

There is a high incidence of MSI in CRC and about 15% of sporadic CRCs progress via an MSI-dependent pathway (30). The testing of MMR/MSI status has been recommended by current guidelines for Lynch syndrome screening in all newly diagnosed CRC and for treatment selection (including chemotherapy and immunotherapy) in metastatic CRC (31, 32). The methods of MMR/MSI detection mainly include IHC, polymerase chain reaction-capillary electrophoresis fragment analysis (PCR-CE), and next-generation sequencing (NGS) panel (33). The CRC patients with dMMR/MSI-high (MSI-H) cannot benefit from fluorouracil-based chemotherapy (34). However, immune checkpoint inhibitor (ICI) therapy, such as programmed cell death protein-1 (PD-1) and programmed cell death ligand-1 (PD-L1) inhibitors, has reversed the treatment landscape and improved survival of these patients in recent studies (35–37). Notably, increasing combination approaches are underway to transform MSS CRC (which accounting for 90-95% of CRC) from the immune desert into immunologically ‘hot’ cancer (38, 39).

Compare to common subtypes of CRC, such as adenocarcinoma and mucinous adenocarcinoma, SRCC is associated with younger on-set (6), aggressive behaviors (7), and a worse overall and disease-free survival (40). Limited whole-exome sequencing and gene panel sequencing profiles demonstrate that general driver mutations associated with CRC (e.g. APC, KRAS, and PIK3CA) are not frequent in SRCC (41, 42). Mutations and signaling pathways predominant in the tumorigenesis of SRCC remains unveiled. Through a Surveillance, Epidemiology, and End Results (SEER) database-based study, Wu et al. concluded that only cancer-specific resection improved the cancer-specific survival in primary colorectal SRCC (8). However, whether surgical removal of breast metastases in patients with limited metastatic disease may confer a survival benefit remains controversial, particularly under the condition of SRCC (25). Prior to metastasectomy, a thorough assessment and systemic treatment should be taken into consideration.

In conclusion, the current case showed that rectal SRCC is associated with younger on-set, aggressive behaviors, and worse survival outcomes. Due to poor cohesiveness, SRCC tends to develop metastases. A patient’s medical history and immunohistochemical staining (such as CK20, CK7, and CDX-2) can aid in identifying the tumor origin of breast and vulvar metastases. Mutations and signaling pathways predominant in the tumorigenesis of SRCC remains unveiled. There is poor effect of conventional chemotherapies, targeted and immunotherapies for colorectal adenocarcinoma on SRCC, so novel therapies are needed to treat this patient population.

## Data availability statement

The original contributions presented in the study are included in the article/supplementary material. Further inquiries can be directed to the corresponding authors.

## Ethics statement

The studies involving human participants were reviewed and approved by Ethical Committee on Biomedical Research, West China Hospital, Sichuan University. The patients/participants provided their written informed consent to participate in this study. Written informed consent was obtained from the participant/patient(s) for the publication of this case report.

## Author contributions

MZ, BZ, YH, and WY conceived and designed the present study. YH and WY drafted the manuscript. QM, ZC, and YY were primarily responsible for literature search and review. JG and TY collected and collated the clinical data. All authors contributed substantially to revising and polishing the manuscript critically for important intellectual content and approved of the final version to be published.
